# Effects of β-Glucans on Pro-Inflammatory Markers and Behavioral Phenotype in a Valproic Acid (VPA)-Induced Autism Model in Zebrafish

**DOI:** 10.3390/ijms27209243

**Published:** 2026-10-18

**Authors:** Diletta Camussi, Maria Marchese, Asahi Ogi, Baldassare Fronte, Ferdinando Nicoletti, Filippo Maria Santorelli, Serena Mero

**Affiliations:** 1 Neurobiology and Molecular Medicine Unit, IRCCS Stella Maris Foundation, 56128 Pisa, Italymaria.marchese@fsm.unipi.it (M.M.);; 2 Department of Clinical Sciences and Translational Medicine, University of Rome Tor Vergata, 00133 Rome, Italy; 3 Department of Veterinary Sciences, University of Pisa, 56126 Pisa, Italy; baldassare.fronte@unipi.it; 4 IRCCS Neuromed, 86077 Pozzilli, Italy

**Keywords:** inflammation, valproate (VPA), zebrafish, β-glucan, oxidative stress

## Abstract

Autism Spectrum Disorder (ASD) is a multifactorial neurodevelopmental condition in which neuroinflammation, oxidative stress, and synaptic dysfunction act as converging mechanisms. Prenatal exposure to valproic acid (VPA), an antiepileptic drug and histone deacetylase inhibitor, is a recognized environmental risk factor associated with ASD-like phenotypes. Zebrafish, with conserved neuroimmune pathways, rapid development, and quantifiable social behavior, represents a robust model for investigating ASD pathophysiology and testing therapeutic candidates. This study aimed to assess if a previously selected concentration of VPA is capable of inducing persistent alterations from early development through adulthood, and to assess the potential of β-glucans, natural polysaccharides with antioxidant and immunomodulatory properties, to mitigate VPA-induced alterations. A multimodal approach included morphological evaluation, mitochondrial respiration, quantitative PCR of inflammatory markers, and behavioral assays targeting locomotion, anxiety-like responses, and social preference. Low-dose VPA (25 μM) induced developmental and behavioral difficulties, accompanied by a transient reduction in mitochondrial respiratory capacity. β-glucan supplementation attenuated selected VPA-induced alterations, including inflammatory gene-expression changes in larvae and behavioral alterations in adulthood. Overall, these findings provide preclinical evidence that β-glucans can modulate selected VPA-induced inflammatory, molecular and behavioral alterations and support further investigation of their potential effects in neurodevelopmental models.

## 1. Introduction

Autism Spectrum Disorder (ASD) is a multifactorial neurodevelopmental condition characterized by deficits in social communication, restricted behaviors, and altered sensory and motor processing [1,2]. Although its prevalence has risen substantially in recent years [3,4], effective treatments targeting core symptoms remain unavailable, underscoring the need for mechanistic studies and novel hypothesis-generating therapeutic strategies.

There is growing evidence that ASD is caused by a combination of a person’s genes and their environment. More than 800 risk-associated loci have been identified, and approximately 100 genes are strongly linked to the development of ASD [5,6]. Many of these genes are involved in the regulation of chromatin remodeling, neurogenesis, synaptogenesis, and inflammatory pathways [7,8,9], which supports the classification of ASD as a disorder rooted in molecular dysregulation. Neuroinflammation and oxidative stress have been proposed as important contributors to ASD among several converging mechanisms. Both autistic individuals and ASD-like animal models exhibit microglial activation. They also exhibit pro-inflammatory cytokine upregulation. In addition, they show chronic immune imbalance within the central nervous system (CNS) [10,11,12]. Microglia are the resident immune cells of the brain. They play essential roles in synaptic pruning, neuronal survival, and plasticity [13,14,15].

Their dysregulation during critical developmental periods may interfere with the maturation and function of neural circuits, thereby affecting brain connectivity and behavior. In parallel, several ASD-associated genes are expressed in brain regions involved in social behavior, including the amygdala [16]. These observations suggest that alterations in neuroimmune signaling may interact with genetic and environmental factors to influence neural circuit development and contribute to ASD-related behavioral difficulties. Thus, neuroimmune dysfunction may represent one of the biological pathways through which genetic susceptibility and environmental exposures converge to affect neurodevelopment.

Among environmental risk factors, prenatal exposure to valproic acid (VPA), an antiepileptic drug and histone deacetylase (HDAC) inhibitor, is one of the compounds most associated with ASD-like phenotypes [17,18]. VPA affects multiple molecular pathways, including GABAergic signaling, ion channel modulation, epigenetic regulation via HDAC inhibition, and mitochondrial metabolism [19,20,21,22,23,24]. These mechanisms converge on synaptic development, neuronal differentiation, and inflammatory signaling, processes known to be altered in ASD [25]. Consequently, VPA exposure during neurodevelopment has been widely used to generate ASD-like phenotypes in animal models [26,27,28,29,30].

Zebrafish have emerged as a powerful system for modeling neurodevelopmental disorders due to their high genetic homology to mammals, easy genetic manipulation, optical transparency, rapid development, and quantifiable social behavior [31,32,33,34,35,36]. VPA-induced zebrafish models recapitulate key ASD-like features, including impaired social interaction, altered locomotion, increased anxiety, and developmental difficulties [37,38,39,40,41,42]. Their suitability for high-throughput screening and in vivo imaging makes them ideal for dissecting molecular mechanisms and testing candidate therapeutics. Despite extensive use of VPA in zebrafish research, the minimal effective dose capable of inducing a stable ASD-like phenotype remains heterogeneous across studies, and the molecular underpinnings, particularly neuroinflammatory and oxidative pathways, require further clarification.

In parallel, attention has been given to β-glucans, that is, natural polysaccharides with antioxidant and immunomodulatory properties, as potential bioactive intervention in multiple disease conditions affecting the brain. β-glucans act as pathogen-associated molecular patterns (PAMPs), with phagocytic cells being activated through pattern recognition receptors (PRRs) and cytokine production being modulated [40,43,44,45,46,47,48,49,50,51,52,53,54]. The capacity to decrease oxidative stress and inflammatory signaling, as well as to regulate microglial activity, has been shown in zebrafish and mammalian models of neuromuscular and neurodegenerative diseases [55,56,57,58,59,60,61,62,63]. These properties suggest that β-glucans may modulate inflammation-driven phenotypes that are relevant to ASD. Importantly, preliminary clinical studies have already explored β-glucan supplementation in children with ASD, reporting changes in behavioral scores, sleep quality and serum melatonin levels, as well as alterations in gut microbiota composition [64]. These findings provide initial clinical support for the potential relevance of β-glucans to ASD-related phenotypes, although the available evidence remains limited and largely based on small pilot studies. However, the effects of β-glucans in experimentally controlled ASD-like models, particularly following VPA exposure, remain insufficiently characterized. In particular, the relationship between early β-glucan exposure, inflammatory and mitochondrial alterations, and behavioral outcomes across developmental stages remains unclear, particularly given the increasing interest in immunomodulatory and antioxidant strategies for neurodevelopmental disorders [65,66,67].

In this work, we aimed to refine the experimental conditions for VPA-induced zebrafish ASD-like phenotypes by identifying a low VPA concentration that produced persistent alterations while preserving sufficient viability for longitudinal analyses, and to investigate the effects of β-glucans across molecular, mitochondrial and behavioral endpoints. We focused on inflammatory signatures, oxidative imbalance, and behavioral deficits, and investigated the potential of β-glucans as an early intervention paradigm. Embryos were exposed to VPA during critical neurodevelopmental windows, and β-glucans were administered 24 h later to evaluate their ability to mitigate inflammation and behavioral difficulties previously identified in our model [68]. Through a multimodal approach integrating molecular, mitochondrial and behavioral analyses, this study provides new insights on the effects of early β-glucan supplementation in a VPA-induced zebrafish model and generates hypotheses regarding the biological pathways that may contribute to the observed phenotypic changes.

## 2. Results

Given the role of CNS neuroinflammation and reactive oxygen species (ROS) production in ASD pathobiology, we evaluated the therapeutic potential of β-glucans across zebrafish development. Larvae at 24 h post-fertilization (hpf) were dechorionated to optimize drug uptake [59]. We then assessed molecular and behavioral markers in both larval and adult zebrafish to determine whether β-glucan exposure could prevent or rescue VPA-induced alterations.

### 2.1. Embryonic and Larval Studies

#### 2.1.1. Morphology

Collected eggs from adult WT-AB zebrafish were randomly assigned to six experimental groups: (1) control, (2) VPA 25 μM, (3) VPA 50 μM, (4) β-glucans 8 mg/L, (5) VPA 25 μM + β-glucans, and (6) VPA 50 μM + β-glucans. The selected concentrations of VPA were administered to the respective experimental groups at 6 hpf, while β-glucans were added at 24 hpf after dechorionation. Embryos were then maintained under these experimental conditions up to 120 hpf.

**Figure 1:**
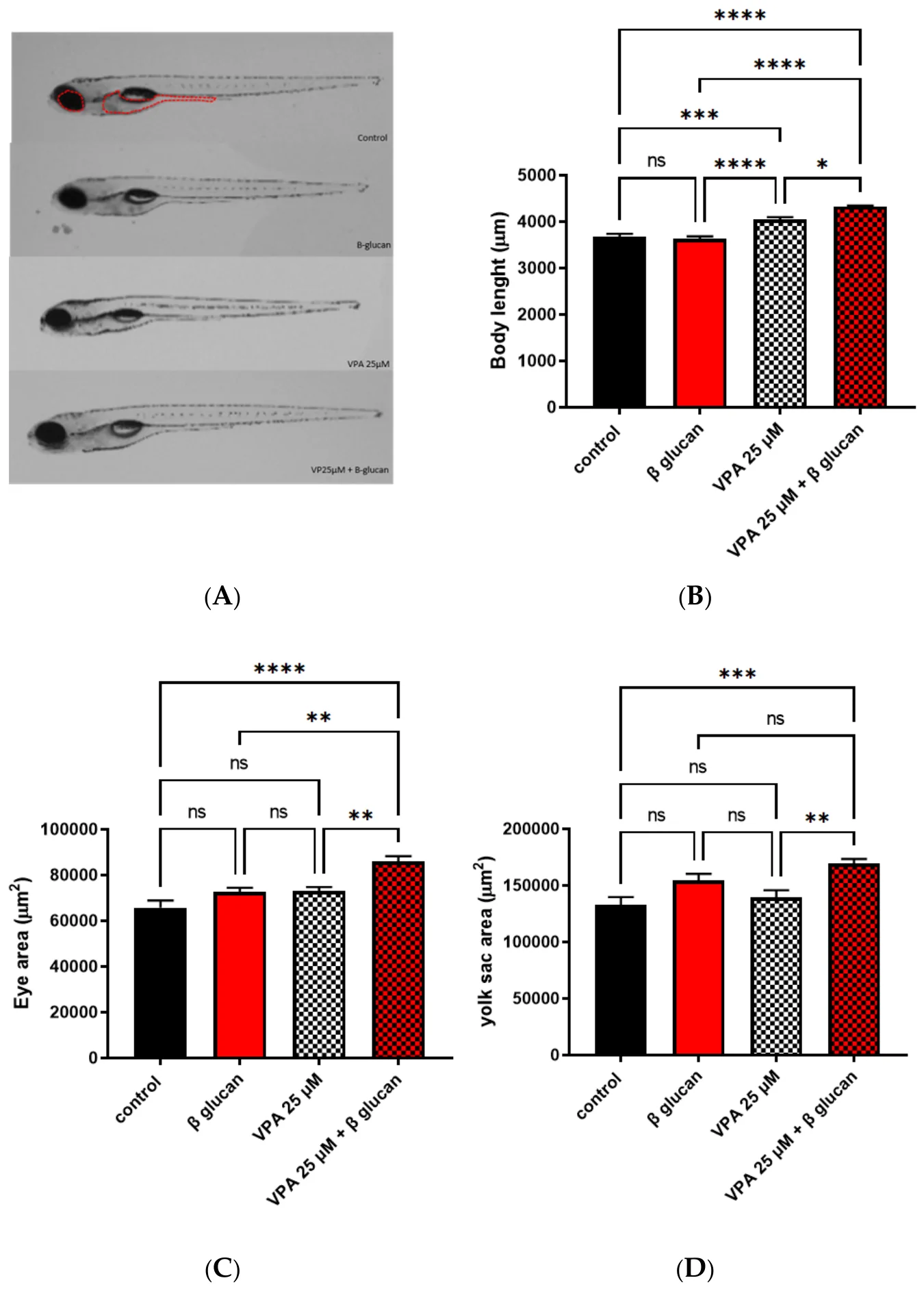
Effects of VPA on larval morphology at 120 hpf (controls and β-glucans 8 mg/L n = 5 per group, VPA-treated larvae and VPA + β-glucan-treated larvae = 10 per group). (**A**) Representative images of WT-AB untreated controls, β-glucan-treated larvae, 25 μM VPA-treated larvae, 25 μM VPA + β-glucans. (**B**–**D**) Morphological evaluation of 25 μM VPA-treated larvae, 25 μM VPA + β-glucans treated fish showed a significant effect (* *p* ≤ 0.05, ** *p* ≤ 0.01, *** *p* ≤ 0.001, **** *p* ≤ 0.0001, ordinary two-way ANOVA with Tukey’s multiple comparisons test) of VPA treatment at 25 μM as compared with untreated controls for eye size, and presence of pericardial edema (yolk sac area) The analyzed ROI were indicated by red dotted lines. Scale bar: 500 μm. Abbreviations: ns = not significant. Data are represented as individual values (lines indicate means ± SEM).

At 50 μM VPA, severe developmental difficulties and toxicity were observed in surviving animals, which substantially limited the interpretability of the phenotypic analyses. Complete mortality subsequently occurred within 15 dpf in both the VPA and VPA + β-glucan groups. Morphological analysis at 120 hpf (Figure 1A) showed that 25 μM VPA was associated with a significant increase in body length, which was not rescued by β-glucans administration (Figure 1B). No difference in the analyzed parameters was detected in the β-glucans-administered group, while a statistically significant increase in eye size and yolk area was measured in the VPA 25 μM + β-glucans group (Figure 1B–D).

#### 2.1.2. Locomotor Behavior

**Figure 2:**
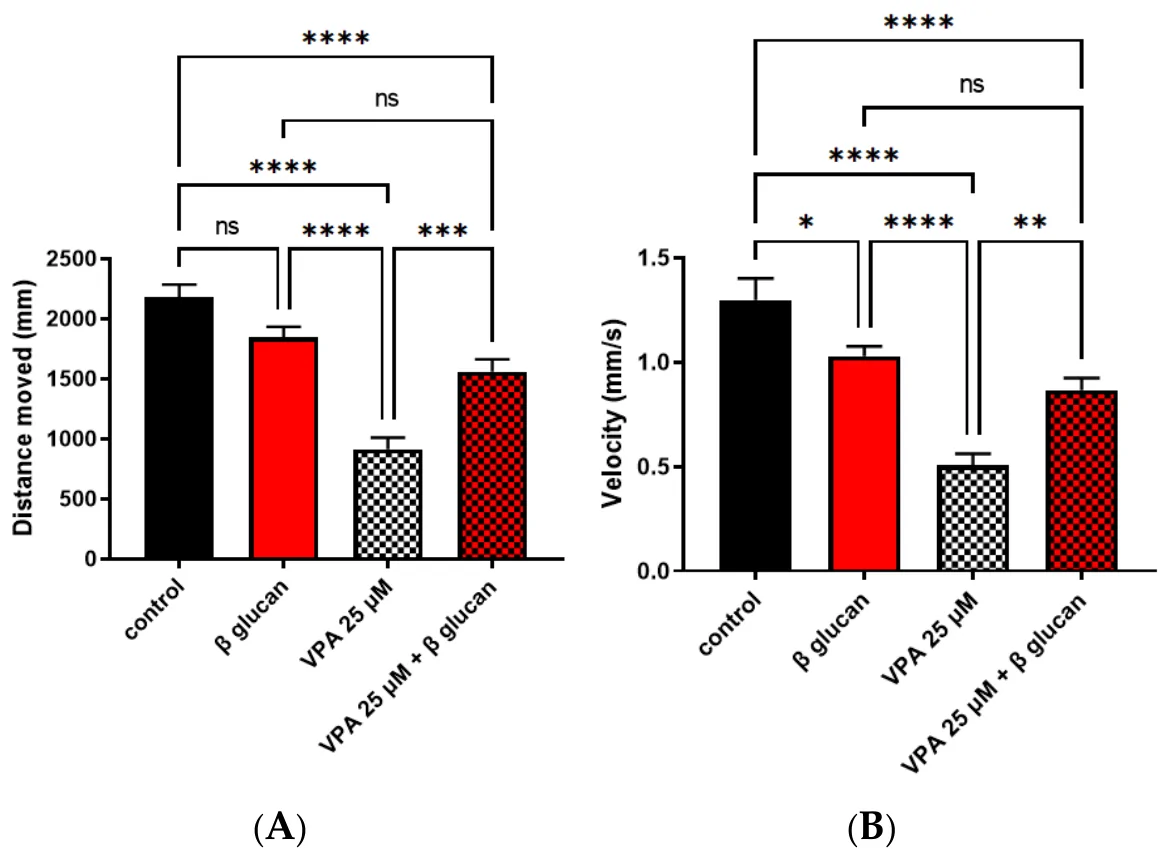
Effects of valproic acid (VPA) on larval locomotor behavior at 120 h post-fertilization (hpf). (**A**,**B**) Distance traveled and velocity were assessed in larvae exposed to 25 μM VPA, with or without β-glucans (8 mg/L). A total of 120 larvae per experimental group were analyzed across five independent experimental runs. For statistical analysis, measurements from individual larvae within each run were averaged to obtain one value per experimental group per run; therefore, n = 5 independent experimental runs per group. VPA exposure significantly reduced locomotor performance, while β-glucan treatment attenuated this effect (* *p* ≤ 0.05, ** *p* ≤ 0.01, *** *p* ≤ 0.001, **** *p* ≤ 0.0001, ordinary two-way ANOVA with Tukey’s multiple-comparisons test). Data are represented as mean ± SEM of the five independent experimental runs.

Based on our previous results [62], 25 μM was identified as an adequate concentration for investigating ASD-like features in our model. Therefore, all subsequent analyses were performed using only the 25 μM VPA exposure. We investigated larval locomotor activity through the tracking analysis, considering two parameters: distance and velocity traveled (Figure 2) in 25 μM VPA-exposed zebrafish. A significant reduction in both parameters was observed in VPA-exposed fish. Overall, treatment with VPA significantly reduced the locomotor performance of the larvae; β-glucans restored locomotor performance in the 25 μM VPA-exposed group (Figure 2A,B).

#### 2.1.3. Analysis of Mitochondrial Respiration

**Figure 3:**
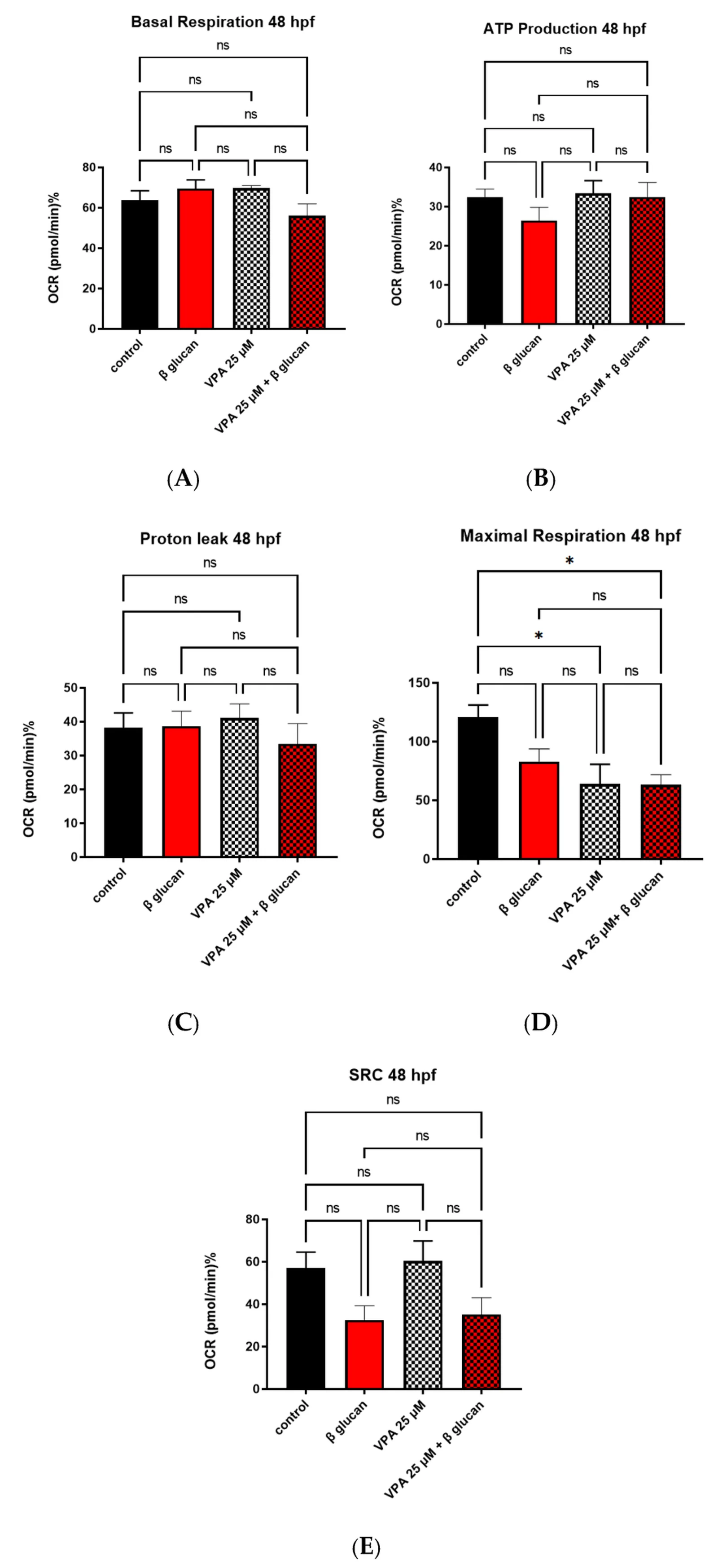
Effects of VPA on WT-AB mitochondrial respiration at 48 hpf. (**A**) Mitochondrial respiratory analysis by basal respiration, (**B**) ATP production, (**C**) proton leak and (**E**) Spare respiratory capacity (SRC) was performed on 12 larvae per experimental group in each experimental run. The experiment was independently repeated twice. Individual larvae within the same experimental run were not considered independent biological replicates. (**D**) A decreased oxygen consumption rate in maximal respiration was detected in VPA-treated groups (* *p* ≤ 0.05, ordinary two-way ANOVA with Tukey’s multiple-comparisons test). Data are represented as mean ± SEM.

In vivo mitochondrial respiration assays revealed mild mitochondrial dysfunction in 25 μM VPA-treated larvae at 48 hpf (Figure 3), whereas no alterations were observed at 120 hpf (Supplementary Figure S1). Specifically, a reduction in maximal respiration rate was detected at 48 hpf, only for VPA-treated groups (Figure 3C). These findings indicate a transient impairment in a single bioenergetic parameter at 48 hpf, suggesting a spontaneous recovery of mitochondrial function by 120 hpf.

#### 2.1.4. Gene Expression Analysis of Inflammatory and Neurodevelopmental Markers

**Figure 4:**
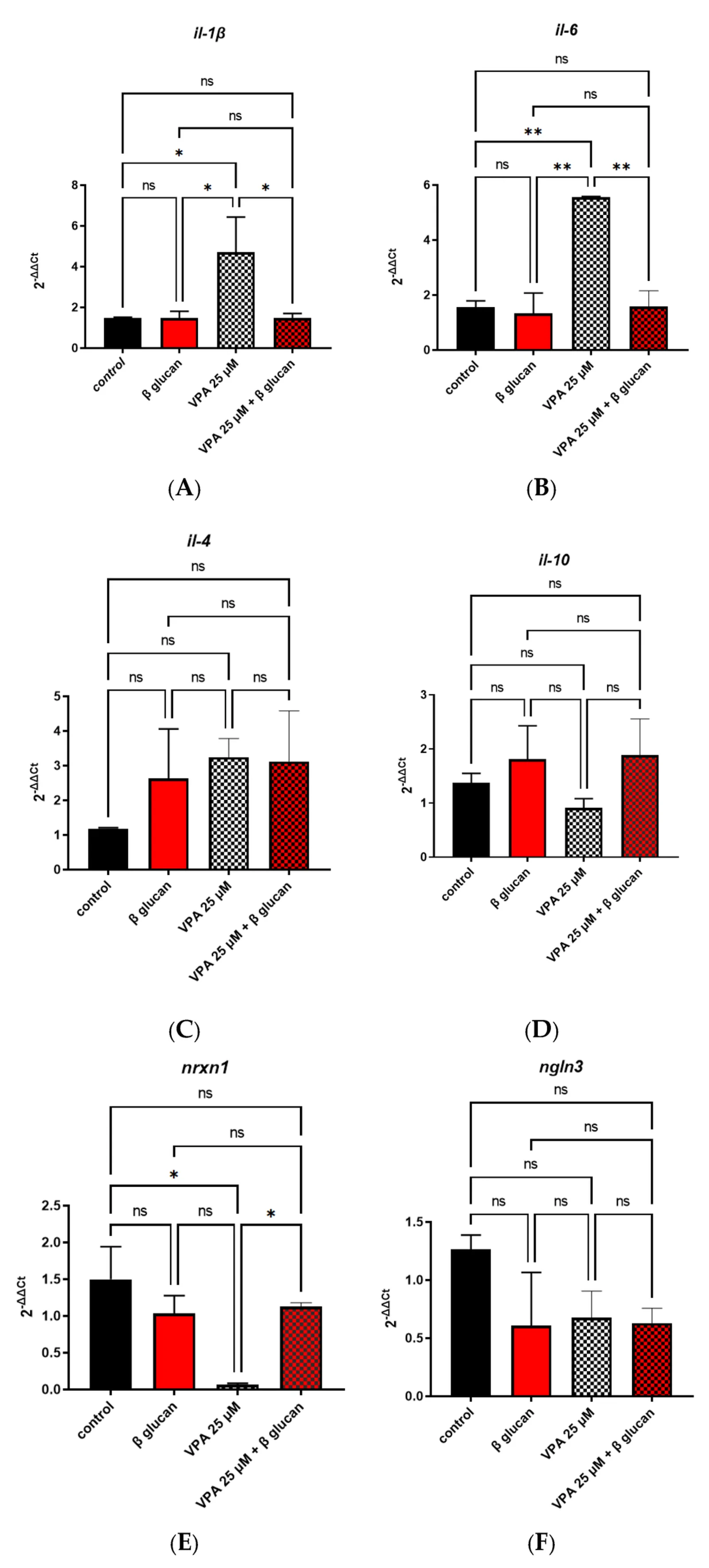
Effects of valproic acid (VPA) on mRNA gene expression in WT-AB larvae at 120 h post-fertilization (hpf). Gene expression was analyzed using three independent biological replicates per experimental group (n = 3), with each biological replicate consisting of a pool of 50 larvae. Technical replicates were performed for each biological sample and were not considered independent observations. Data are represented as mean ± SEM. (**A**,**B**) Data analysis showed a significant upregulation of pro-inflammatory cytokines *(il*-6 and *il*-1*β*) in 25 μM VPA-treated larvae compared with controls, and a restored inflammatory profile by the administration of β-glucans (* *p* ≤ 0.05, ** *p* ≤ 0.01, ordinary two-way ANOVA with Tukey’s multiple comparisons test). (**C**,**D**) No significant difference was detected in the expression of the anti-inflammatory cytokines (*il*-4 and *il*-10). (**E**,**F**) The neurogenesis-related gene, *nrxn*1, was downregulated in 25 μM VPA-treated larvae compared with controls, and its expression was restored by the administration of β-glucans (* *p* ≤ 0.05, ordinary two-way ANOVA with Tukey’s multiple comparisons test). No difference has been detected in the expression of Neuroligin-3 (*ngln*3) in treated larvae versus controls. Data are represented as means ± SEM. Abbreviations: ns = not significant. Primer available in Supplementary Material (Table S1).

Gene expression analysis performed on 120 hpf larvae of all four groups (Figure 4) showed an increased pro-inflammatory profile, with augmented expression of cytokines interleukin-1 beta (*il*-1*β*) and interleukin-6 (*il*-6); this was attenuated following the administration of β-glucans (Figure 4A,B). A statistically significant downregulation of the neurodevelopment-related gene *nrxn*1 was observed following exposure to 25 μM VPA, and this effect was again attenuated by β-glucan treatment (Figure 4E). However, there were no significant differences in the expression of the anti-inflammatory markers IL-10 and IL-4, or of the neurodevelopment-related gene nlgn3, following VPA exposure or subsequent β-glucan treatment (Figure 4C,D,F).

### 2.2. Behavior Analyses on Adult Treated Fish

**Figure 5:**
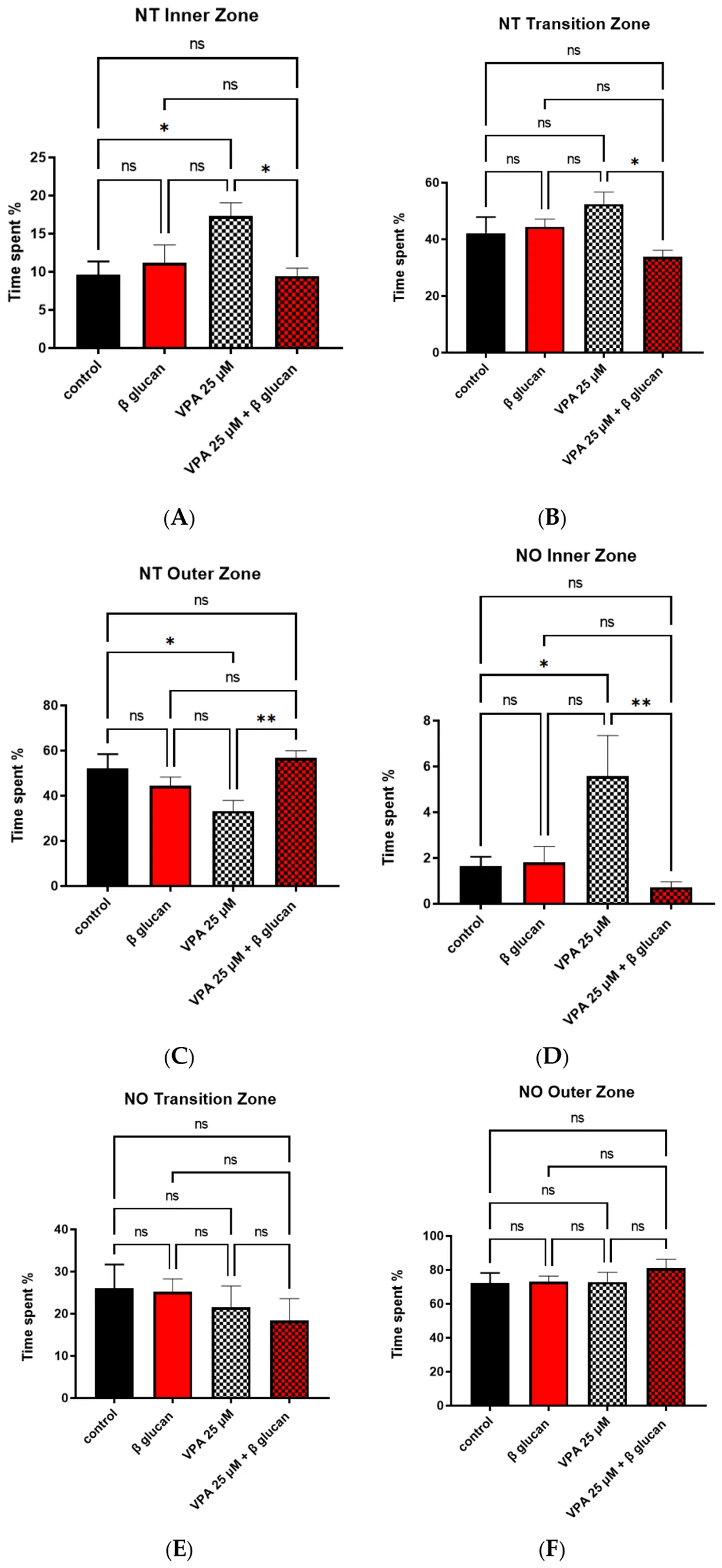
Novel Tank and Novel Object Exploration test performed on adult WT-AB zebrafish (4 mpf) exposed for 120 hpf to VPA 25 μM, β-glucans and VPA 25 μM + β-glucans. (**A**–**C**) Novel Tank behavioral test on 4 mpf WT-AB zebrafish: VPA-only treated group (n = 10), VPA 25 μM + β-glucans (n = 10), VPA 25 μM, β-glucans (n = 10), and control (n = 10). Inner zone (* *p* ≤ 0.05, ordinary two-way ANOVA with Tukey’s multiple comparisons test), intermediate zone, and outer zone (* *p* ≤ 0.05, ** *p* ≤ 0.01, ordinary two-way ANOVA with Tukey’s multiple comparisons test). (**D**–**F**) Novel Object Exploration test in 4 mpf WT-AB zebrafish: VPA-only treated group (n = 10), VPA 25 μM + β-glucans (n = 10), VPA 25 μM only, β-glucans only, and control group (each n = 10). Inner zone (* *p* ≤ 0.05, ** *p* ≤ 0.01, ordinary two-way ANOVA with Tukey’s multiple comparisons test), intermediate zone, and outer zone. Abbreviations: ns = not significant; NT=Novel Tank; NO=Novel Object. Data are represented as means ± SEM.

We conducted a Novel Tank test [69] on 4 months post-fertilization (mpf) adult zebrafish exposed to 25 μM VPA and 25 μM VPA + β-glucans. A statistically significant increase in time spent in the inner zone of the tank and a decrease in time spent in the outer zone of the tank were detected for VPA-exposed group; the administration of β-glucans reverted the phenotype to the control group levels (Figure 5A–D). The Novel Object Recognition test [70] conducted across all four experimental groups revealed that fish exposed to VPA alone spent significantly more time exploring the novel object compared to the other groups, whereas no statistically significant differences were observed regarding the time spent in the outer and transition zones (Figure 5E,F). Furthermore, a social preference test was conducted, but the analysis of data did not show a statistically significant difference between the treated and control groups (Supplementary Figure S2A).

## 3. Discussion

ASD is a neurodevelopmental disorder characterized by persistent impairments in social communication and restricted, repetitive patterns of behavior [1]. It affects a considerable proportion of the global population, with a prevalence ranging from approximately 1 in 36 to 1 in 77 individuals depending on the population studied [71]. Given its high prevalence and the profound health-related social impact on the quality of life of affected individuals and their families, further investigation into the biological mechanisms underlying ASD, as well as the development of effective therapeutic strategies, remains a priority.

Over the past decades, animal models have played a pivotal role in elucidating the complexity of pathophysiological mechanisms associated with ASD and in identifying potential therapeutic targets. Among the available models, zebrafish have gained increasing value due to their genetic and neurobiological similarities to mammals, along with the conservation of several behavioral traits relevant to ASD. In addition, zebrafish offer several experimental advantages, including rapid external development and high genetic manipulability, making them suitable for generating disease models [72]. Despite the numerous advantages of using zebrafish as a model for ASD, several important limitations should be considered. First, VPA acts as a broad-spectrum teratogen causing widespread neurodevelopmental alterations rather than selectively targeting ASD-relevant pathways, which may partially interfere with the construct validity of the model [73,74]. Furthermore, behavioral and molecular outcomes are highly sensitive to VPA dose, developmental stage at exposure, and environmental conditions, resulting in considerable variability across studies and laboratories [73,75]. Finally, VPA exposure can induce morphological difficulties that may confound the interpretation of locomotor, sensory, and social behavioral assays [74].

Previous investigations have demonstrated that embryonic exposure to increasing concentrations of VPA during critical developmental windows successfully reproduces several important features of ASD observed in humans, including challenges in social behavior and neurobiological difficulties [74,75,76,77,78]. In our study, we sought to validate a zebrafish ASD-like model in 5 dpf larvae and to better elucidate the role of neuroinflammation and microglial alteration on pathophysiology of ASD [62]. We conducted a systematic review focusing exclusively on behavioral outcomes in adult zebrafish exposed to VPA [77]. The review revealed a paucity of studies that specifically investigated adult behavioral phenotypes, and it showed a great deal of heterogeneity in the VPA concentrations that were used, making it very difficult to make direct comparisons between studies. However, the evidence suggests that concentrations of 25 μM and 48 μM are the lowest effective doses capable of inducing an ASD-like phenotype. Building upon these findings, the present study was designed not to establish a new VPA-induced zebrafish model, but to use an experimentally defined VPA exposure paradigm to investigate the effects of early β-glucan supplementation on VPA-induced molecular, mitochondrial and behavioral alterations. Because β-glucans were administered at 24 hpf while VPA exposure continued until 120 hpf, this experimental design represents an early intervention during ongoing VPA exposure rather than treatment of an established ASD-like phenotype. In this context, we investigated the inflammatory and oxidative-related profile in our VPA-induced ASD-like model and evaluated the potential modulatory effects of β-glucans, in line with studies performed in zebrafish models of neuropsychiatric disorders [57]. Among others, β-glucans have emerged as particularly promising owing to their well-documented immunomodulatory and antioxidant activities [59]. β-glucans are a group of natural polysaccharides, displaying numerous non-toxic properties and bioactive functions [43,53,79] and displaying immunomodulatory function through the enhancement of the activity of phagocytic cells [47]. Beyond their enhancer role, β-glucans trigger numerous intracellular processes, including the regulatory mechanism of ROS production and inflammation-related cytokines [49]. Moreover, probably in relation to their intrinsic structure, they can remove free oxygen radicals in vivo [50,51,52]. In this work, zebrafish embryos were exposed to either 25 μM or 50 μM VPA following the protocol established in our previous study [62]; to maximize β-glucan uptake, embryos were dechorionated at 24 hpf, a procedure previously shown to improve compound absorption [57]. Because of this experimental change, key morphological, behavioral, and molecular analyses were repeated during both larval development and adulthood.

Initially, we characterized the morphological and behavioral phenotype during larval development. The VPA paradigm in this study was restricted to two concentrations (25 and 50 µM). The higher dose resulted in complete mortality between 5 and 15 dpf regardless of β-glucan treatment, thereby preventing a comprehensive dose–response characterization. Our data suggest that 50 µM VPA elicits severe developmental toxicity rather than a specific ASD-like phenotype. Consequently, the identification of 25 µM as the ‘minimal effective dose’ must be interpreted cautiously, awaiting further studies on a broader dose–response profile.

Larvae exposed to 25 μM VPA displayed morphological difficulties, including body length, which remained unaffected by β-glucan administration. These structural difficulties may influence locomotor and sensory performance and therefore represent potential confounders for the interpretation of ASD-like behavioral outcomes. Consistent with our previous observations, VPA exposure significantly reduced locomotion by impacting both swimming distance and velocity at 120 hpf. Remarkably, β-glucan treatment partially restored locomotor performance in larvae exposed to 25 μM VPA. These findings are consistent with previous reports demonstrating that β-glucans effectively improve early motor impairments in other zebrafish disease models [57,80]. We also performed microscopical and in vivo mitochondrial respiration assays at two developmental time points, namely, 48 hpf and 120 hpf. The experiment showed a significant reduction in maximal respiration rate in VPA-treated larvae at the earlier time point, indicative of mild impairment of mitochondrial function. However, this challenge was not rescued by the β-glucan administration. Interestingly, by 120 hpf, mitochondrial respiration appeared to normalize, suggesting a spontaneous recovery of oxidative capacity. A selective reduction in maximal respiration rate, without affecting basal OCR, indicates an impairment of mitochondrial respiratory capacity rather than baseline ATP collapse [62,79]. This pattern may reflect a mild bioenergetic vulnerability where mitochondria can supply a resting energetic demand but fail under acute stress [79]. Previous studies have reported that VPA can induce transient oxidative stress and mild, reversible inhibition of respiratory complexes I and IV [62,72]. The normalization of the maximal respiration rate parameter by 120 hpf likely reflects a compensatory mechanism during zebrafish development [78,79]. According to the previous literature [62,72,79], this metabolic recovery could be driven by several synergistic physiological processes involving developmental and bioenergetic aspect: (i) rapid organelle maturation upregulates biogenetic pathways between 48 and 120 hpf to offset early deficits [68]; (ii) VPA-induced acute damage triggers a quality control mechanism in mitochondria (such as mitophagy) that clears damaged mitochondria by 120 hpf [81], or a combination of the two. Furthermore, the maturation of metabolic organs such as the liver, which takes place between 72 and 96 hpf, enhances endogenous antioxidant defenses [78,79], allowing for a transcriptional and metabolic realignment that resolves the transient developmental energetic impairment induced by early VPA exposure [62,72].

We subsequently examined the expression of selected inflammatory and neurodevelopmental-related genes. Consistent with the reported anti-inflammatory properties of β-glucans [80], treatment normalized the VPA-associated upregulation of the pro-inflammatory cytokine genes *il*-1*β* and *il*-6, [82]. In contrast, no significant changes were detected in the expression of the anti-inflammatory cytokines *il*-4 and *il*-10 or in *nlgn*3 following VPA exposure. This finding may reflect the distinct temporal dynamics of the inflammatory response, as pro-inflammatory cytokines are rapidly induced following immune activation, whereas anti-inflammatory mediators are generally involved in the subsequent resolution phase to suppress excessive inflammation and restore tissue homeostasis [82,83]. Therefore, at the analyzed developmental stage (120 hpf), the anti-inflammatory response may still display a different temporal profile from the early induction of pro-inflammatory cytokines. Alternatively, the absence of changes in *il*-4 and *il*-10 may itself reflect the immunomodulatory rather than purely anti-inflammatory activity of β-glucans. Indeed, β-glucans are recognized by pattern recognition receptors, including Dectin-1, leading to the modulation of signaling pathways such as NF-κB that limit excessive production of pro-inflammatory mediators while preserving immune homeostasis, rather than directly inducing anti-inflammatory cytokine expression [84,85]. Consequently, by dampening the initial inflammatory response, β-glucan treatment may have reduced the need for a compensatory induction of *il*-4 and *il*-10, maintaining their expression at basal levels. Regarding neurodevelopmental-related gene expression, β-glucan administration fully restored the expression of *nrxn*1, suggesting a potential modulatory effect on neurodevelopmental gene expression. In contrast, no significant changes were observed in *nlgn*3 expression. Although neurexins and neuroligins function as trans-synaptic adhesion partners, increasing evidence indicates that they are regulated through distinct molecular mechanisms. Neurexins are presynaptic organizers whose expression is highly responsive to neuronal activity, synaptic maturation, and environmental perturbations, making nrxn1 particularly vulnerable to neurodevelopmental insults. Conversely, neuroligins are postsynaptic adhesion molecules whose expression is more tightly regulated during synapse maturation and can be partially compensated by the functional redundancy among neuroligin family members [86,87]. Thus, the restoration of *nrxn*1 expression by β-glucan may reflect the preservation of presynaptic organization and synaptic integrity, whereas the unchanged *nlgn*3 expression suggests that postsynaptic adhesion complexes were either less affected by VPA exposure or maintained through compensatory regulatory mechanisms [86].

Previous clinical studies have explored the potential effects of β-glucan supplementation in individuals with ASD. Small pilot studies using a β-1,3/1,6-glucan preparation derived from *A. pullulans* reported preliminary improvements in behavioral scores and sleep quality, together with changes in serum melatonin levels [64,88]. A subsequent pilot study also reported changes in gut microbiota composition following β-glucan supplementation [89]. These findings provide preliminary clinical evidence that β-glucans may influence biological and behavioral parameters relevant to ASD. However, the available clinical evidence remains limited by the small sample sizes and exploratory nature of these studies, and does not establish the molecular mechanisms underlying the observed effects. In this context, our study provides complementary preclinical evidence by examining β-glucan effects in a controlled VPA-induced zebrafish model and by integrating behavioral analyses with mitochondrial respiration and inflammatory and neurodevelopmental gene expression. Thus, the contribution of the present study should not be interpreted as demonstrating a novel therapeutic property of β-glucans per se, but rather as providing experimental evidence on their effects within a defined ASD-like developmental model and generating hypotheses regarding the biological pathways that may contribute to these effects.

Despite the potential significance of these findings, some limitations should be acknowledged. First, the observed effects on mitochondrial respiration were transient, and further functional rescue experiments and direct oxidative measurements are required to confirm the underlying mechanisms. Secondly, the molecular profiling was confined to a limited array of targets and was conducted on entire larvae, impeding our comprehension of tissue-specific responses. Therefore, these findings cannot be interpreted as direct evidence of brain-specific neuroinflammation or microglial activation. Likewise, oxidative stress was not directly quantified in the present study. Third, we confined the β-glucan administration regimen to a single dose and a unique timing window, limiting our ability to determine whether different doses or treatment windows would produce distinct effects. To fully understand the potential of this approach, future studies should include broader molecular screening, tissue-specific analyses, and a multi-dose regimen across different life stages.

Finally, we assessed whether the beneficial effects of β-glucans extended into adulthood by carrying out behavioral analyses in zebrafish previously exposed to 25 μM VPA during embryonic development. To evaluate these effects in our ASD-like zebrafish model, adult fish underwent the Novel Tank test and the Novel Object Exploration test. In both tests, β-glucan supplementation completely reversed the behavioral alterations induced by VPA exposure. Specifically, in the Novel Object Exploration test, VPA-treated fish exhibited altered exploration of the inner zone, a behavioral phenotype that was fully normalized following β-glucan treatment. Collectively, these findings may suggest that VPA induces an altered anxiety-like and novelty-directed behavioral profile, characterized by increased boldness, exploration or reduced risk assessment. Finally, social behavior was evaluated using the social preference test; although VPA-exposed fish showed a trend towards altered social preference, this difference did not reach statistical significance. Adult behavioral assessment in the present study was limited to the Novel Tank and Novel Object Exploration Tests, which primarily capture anxiety-like responses, exploration and boldness. Although these paradigms are relevant to ASD-associated behavioral dimensions, they do not provide a comprehensive evaluation of complex social and cognitive functions. Accordingly, the behavioral alterations observed in these assays should be interpreted as changes in anxiety-like, exploratory and novelty-related responses rather than as direct evidence of cognitive impairment or a specific ASD behavioral domain.

Moreover, the social preference assay did not reveal statistically significant differences between groups, suggesting that potential alterations in social behavior would require further confirmation using additional dedicated social paradigms and larger sample sizes. Accordingly, interpretations related to “cognitive dysfunction” and social impairment in adulthood are hypothesis-generating and confined to the specific behavioral domains assessed here. Indeed, limitations include small sample size in adult behavioral assays (n = 10 per group) and mitochondrial respiration (n = 12 per group), and sex was not controlled as a biological variable in adult zebrafish—all factors which may have limited our ability to detect subtle effects.

The behavioral normalization observed following β-glucan supplementation supports further investigation of its potential effects on VPA-induced behavioral alterations.

Finally, all these findings suggest that early VPA exposure induces multiple, interconnected but partially independent alterations involving mitochondrial function, inflammatory signaling, and neuronal pathways. The normalization of selected inflammatory and molecular alterations following β-glucan supplementation, together with the recovery of some aspects of behavioral phenotype, is consistent with a potential contribution of these pathways to selected long-term behavioral outcomes. However, the persistence of other phenotypes indicates that inflammatory alterations alone are unlikely to account for the entire behavioral phenotype. Early mitochondrial dysfunction may represent an additional factor associated with the functional difficulties observed following VPA exposure. Thus, the lack of a uniform relationship between cytokine changes and behavioral outcomes may reflect the multifactorial nature of VPA-induced neurodevelopmental alterations.

## 4. Materials and Methods

### 4.1. Ethical Regulation

The experimental procedures were conducted under the supervision and with the approval of the Italian Ministry of Health (approval code: n° 14/2026-PR; approval date: 13 January 2026) and Use Committee of the IRCCS Stella Maris Foundation (Pisa, Italy) and in compliance with European Directive No. 63 of 22/09/2010 on the protection of animals used for scientific purposes. Throughout the study, zebrafish embryos and larvae up to 5 days post-fertilization (dpf) were used, in line with the “3Rs” guiding principles for research involving the use of laboratory animals [90].

### 4.2. Chemicals

Valproic acid sodium salt (CAS-No: 1069-66-5) and tricaine (CAS-No: 886-86-2) were obtained from Sigma-Aldrich (St. Louis, MO, USA). A 100 mM stock solution of VPA was prepared by dissolving VPA in ultra-pure water. Experimental solutions were freshly prepared in standard fish medium, which consisted of osmotic water with 60 mg per liter of “Instant Ocean^®^” sea salt (Spectrum Brands, Blacksburg, VA, USA). Drug doses were chosen based on preliminary results and data obtained in our previous work [62], ranging from 25 to 50 μM. At 24 hpf, butches of 40–50 embryos were incubated for 30 min in 1 mg mL^−1^ Pronase^®^ (Sigma-Aldrich, Steinheim, Germany) solution. Afterwards, embryos were rinsed three times for 3 min with egg water [90]. Subsequently, viable eggs were distributed in 5.5 cm diameter Petri dishes containing 10 mL of egg water each. The treatments consisted of 3–4 replicates and 20–30 eggs per replicate (according to egg availability). Before to perform the treatment, a 20 mg mL^−1^ stock suspension of 1,3–1,6 β-glucans extracted from Saccharomyces cerevisiae cell wall (MacroGard^®^, Biorigin©, São Paulo, Brazil) was prepared and then added to the Petri dishes to obtain the β-glucans concentration of 8 mg L^−1^, which was, hence, administered to dechorionated embryos, on the base of the previous literature [57,90].

### 4.3. Animals

Adult wild-type AB strain (WT-AB) zebrafish from the Department of Neurobiology and Molecular Medicine of the IRCCS Stella Maris Foundation were used. Parental fish were maintained according to standard procedures [91], and once spawned, eggs were collected and incubated at 28.5 °C in Petri dishes (Ø 10 cm, 40 eggs per Petri) filled with 50 mL of egg water.

The experiments were conducted on WT-AB zebrafish larvae (2 to 5 dpf according to the experiment) and on adult WT-AB zebrafish (4-month-old) maintained at the animal facility of the IRCCS Stella Maris Foundation. Adult fish were housed in a recirculating aquaculture system (RAS) equipped with 3.5 L tanks (Tecniplast S.p.A., Buguggiate, Italy), water temperature was set at 28.5 °C, and a 14 h light/10 h dark cycle was applied, according to standard procedures [90,91]. Dechorionated zebrafish larvae were divided into six experimental groups: (1) untreated controls (control), (2) 25 μM VPA-treated larvae (VPA 25 μM), (3) 50 μM VPA-treated larvae (VPA 50 μM), (4) β-glucan-treated larvae (8 mg/L) (β glucans), (5) larvae treated with combination of 25 μM VPA and β-glucans (VPA 25 μM + β-glucans), and (6) larvae treated with combination of 50 μM VPA and β-glucans (VPA 50 μM + β-glucans), where VPA and β-glucans were administered at 6 hpf and 24 hpf respectively, up to 120 hpf. Morphological, bioenergetic, molecular and locomotor analysis were carried out at 120 hpf. Larvae were allowed to grow up to 4 mpf and behavioral tests of Novel Tank–Novel Object and social preference were performed. In this study, blinding was not applied during the experimental procedures as the researchers were aware of the group assignments. However, randomization was employed to minimize potential bias in the allocation of experimental units. Embryos were randomly assigned to control and treatment groups by drawing lots, ensuring that allocation was not based on embryo characteristics or experimental outcome. Potential confounding factors related to the order of treatments and measurements or the position of experimental units were considered. Given the experimental design, in which zebrafish embryos/larvae were individually allocated and treated under standardized conditions, these factors were not expected to substantially influence the experimental outcomes.

### 4.4. Morphological Evaluation

At 120 hpf, all groups underwent morphological evaluation, which included measurements of body length, yolk area and eye size. Larvae were first anesthetized by immersion in a water bath containing tricaine (0.168 mg/mL) and then individually placed in a transparent cavity slide under the Leica M205FA stereomicroscope for image capture. Data analysis was performed using DanioScope software 2.1.

### 4.5. Test of Larval Locomotor Behavior

The locomotor behavior analysis included an assessment of velocity and distance movement. Larval locomotion was analyzed for 20 min. The test was performed on WT-AB hatched larvae at 120 hpf using 96-well plates (1 larva per well in 300 μL of medium) placed in the DanioVision apparatus connected to EthoVision XT17 software (Noldus Information Technology, Wageningen, The Netherlands).

### 4.6. Mitochondrial Respiration Assay

Mitochondrial respiration analysis was conducted following the protocol outlined by Naef et al. [92]. The assay was performed at 48 hpf and 120 hpf using the XF24 extracellular flux analyzer (Seahorse Bioscience, North Billerica, MA, USA).

### 4.7. Quantitative Reverse Transcription Polymerase Chain Reaction (qRT-PCR)

Total RNA was extracted from 30 larvae at 120 hpf per group using Quick RNA miniprep (Zymo Research, Irvine, CA, USA) according to the manufacturer’s instructions. cDNA synthesis and qRT-PCR analysis were performed as described by Licitra et al. [91]. To quantify relative mRNA expression, the Mic Real-Time PCR System (Bio Molecular Systems, Upper Coomera, Australia) and the comparative ΔCt method were used. All data were normalized to the expression of the housekeeping gene, β-actin. Supplementary Table S1 lists the sequences of the primers used.

### 4.8. Test of Adult Behavior

Standardized behavioral tests were used to assess various aspects of anxiety and social interaction in adult zebrafish. All tests were performed in tanks filled with RAS water maintained at a constant temperature of 28.5 °C by using a heating mat and under controlled lighting conditions. The animals were tested after their usual morning feeding to prevent stress from food deprivation. The tanks of fish to be tested were kept for two hours in the test room to allow the animals to acclimatize. In detail, for the Novel Tank and Novel Object Exploration Tests, a 1.7 L tank (10.6 cm high × 20 cm wide × 12.5 cm long; Tecniplast S.p.A., Buguggiate, Italy) was used. Social preference was performed using 5 compartments of a Noldus T-maze (4 L). Each compartment is a transparent cube of 10 cm where it is possible to insert transparent dividers.

#### 4.8.1. Novel Tank Test and Novel Object Exploration Test

The Novel Tank (NT) or Open Field test was conducted to evaluate the individual’s response to a novel environment, where fish typically exhibit swimming behavior by spending less time in the inner zone of the tank due to their natural tendency to display thigmotactic behavior, in terms of time spent in the inner, transition or outer zone. In zebrafish, thigmotaxis is the instinctive “wall-hugging” behavior of staying close to the boundaries of an environment and is considered a validated measure of anxiety, where a preference for the edges of a tank indicates increased anxiety or stress, while reduced thigmotaxis suggests lower anxiety and greater exploration of the environment [93]. Single zebrafish were introduced into a novel tank, and behavior was video recorded over a 10 min observation period. The tank was virtually divided into three concentric zones: inner, middle, and outer. Distance moved, velocity, and time spent in each zone were measured for each tested fish to evaluate their swimming activity and zone preference in an unfamiliar place. The Novel Object (NO) test assesses zebrafish’s exploratory behavior towards novel objects. Zebrafish are naturally curious and tend to explore novel objects more than familiar ones. Reduced exploration of the novel object can indicate anxiety or impaired cognitive function [88]. After the Novel Tank test registration, a novel object, consisting of a colored Lego^®^ brick (2 × 4 dots, 8 mm high), was introduced into the center of the test arena, before tracking started again. The fish was recorded over 10 min more of observation period and this allowed for an evaluation of the fish’s anxiety levels towards a novel object. The Novel Object (NO) test was performed following the apparatus and procedural sequence described by Johnson et al. [94]. Readers are referred to the original publication for a comprehensive description of the arena design 19.

#### 4.8.2. Social Preference Test

The social preference test (Supplementary Figure S2B) enables the assessment of fish sociality through the observation of their interactions with conspecifics [66]. Each tested fish was placed in the central compartment of a T-maze (Noldus Information Technology) and allowed to explore the new environment for 30 s. Subsequently, in one of the two side compartments, a group of 4 zebrafish of the same age and size (social stimulus) were placed, whereas the other was left empty (no social stimulus). The time spent near the compartment with social stimulus compared to the empty compartment was recorded for an additional 6 min. An increased duration spent near the compartment with conspecifics indicates a preference for social interaction, while a preference for the empty side suggests individual social impairment. The compartment containing the social stimulus was alternated with the empty one between one test and another to avoid behavioral bias.

### 4.9. Statistical Analysis

Sample sizes were determined based on biological feasibility, experimental variability, and sample sizes reported in previous studies using comparable zebrafish models and assays. No formal a priori sample-size calculation was performed. Data distribution was assessed using the Shapiro–Wilk test. For experiments following a 2 × 2 factorial design, with VPA exposure and β-glucan treatment as the two factors, an ordinary two-way ANOVA was used to evaluate the main effects of VPA exposure, β-glucan treatment, and their interaction, followed by Tukey’s multiple-comparisons test for post hoc comparisons. This approach was applied to the relevant morphological, locomotor, mitochondrial respiration, gene-expression, and adult behavioral analyses. Statistical significance was defined as *p* ≤ 0.05, and all analyses were performed using GraphPad Prism 9 (GraphPad Software, San Diego, CA, USA).

For gene-expression analyses, larvae were collected as pooled biological samples. Each biological replicate consisted of a pool of 50 larvae, and three independent biological replicates were performed for each gene and experimental group. Technical replicates were performed for each biological sample and were not considered independent biological replicates.

For locomotor analyses, a total of 120 larvae per experimental group were analyzed across five independent experimental runs. Within each run, measurements from individual larvae were averaged to obtain one value per experimental group. These run-level values were used as the independent observations for statistical analysis (n = 5 independent experimental runs per group), thereby avoiding pseudoreplication of individual larvae originating from the same experimental run.

For mitochondrial respiration, 12 larvae per group were analyzed across two independent experimental runs. Individual larvae within the same experimental run were not considered independent biological replicates. The experiment was independently repeated twice.

For adult behavioral experiments, 10 fish per group were analyzed. The sex of adult fish was not recorded and therefore was not included as an experimental factor.

Embryos were randomly allocated to the experimental groups to minimize potential allocation bias. No animals or experimental units were excluded because of experimental procedures or technical failure. Statistical outliers were identified using Grubbs’ test in GraphPad Prism 9.0 using a significance threshold of α = 0.05. When an observation was identified as a statistical outlier according this predefined criterion, it was excluded from the corresponding statistical analysis. Outlier identification and exclusion were performed independently of the experimental group comparison and were not based on whether exclusion affected the statistical significance of the results. All statistical analyses were performed on the resulting datasets after application of the predefined outlier criterion.

## 5. Conclusions

In conclusion, this paper provides a comprehensive insight into the neurodevelopmental and behavioral effects of VPA exposure in zebrafish lifespan, highlighting its role in modeling ASD-like phenotypes. The assessment of a wide range of behavioral and molecular parameters highlighted the impacts of VPA on embryonic development, supporting that VPA-induced alterations in neurodevelopmental processes may exert important implications for a deeper knowledge of the mechanisms driving the pathogenesis of ASD. We also evaluated their persistence over time. Our behavioral analyses confirmed their persistence, with pronounced impairments in exploratory behavior observed in VPA-treated groups. Within an early co-exposure paradigm, and guided by our previous results, we selected β-glucans as a candidate compound to counteract the pro-inflammatory state characteristic of this model. Consistent with evidence from other disease models, β-glucans demonstrated notable efficacy in mitigating the proinflammatory environment induced by early VPA exposure—both at the behavioral and molecular levels in larvae, and to a certain extent at the behavioral level in adulthood. Collectively, these findings support the therapeutic potential of β-glucans in alleviating ASD-like behavioral phenotypes resulting from early developmental exposure to VPA.

This work offers a solid background for future work into the potential of anti-inflammatory compounds in preventing and attenuating the self-perpetuating processes underlying ASD and other neurodevelopmental disorders, within a broader translational perspective.

It would be useful to explore alternative timing for β-glucan administration and to assess the molecular effects of β-glucan in terms of CNS gene expression in adulthood. This would provide additional data to correlate with the effects of anti-inflammatory or otherwise beneficial therapies on both social and anxiety-related behaviors, especially in response to novelty.

## Data Availability

The original contributions presented in the study are included in the article/Supplementary Materials; further inquiries can be directed to the corresponding authors.

## Supplementary Materials

The following supporting information can be downloaded at https://www.mdpi.com/article/10.3390/ijms27209243/s1.
