# Supplementary material for: Effects of β-Glucans on Pro-Inflammatory Markers and Behavioral Phenotype in a Valproic Acid (VPA)-Induced Autism Model in Zebrafish
Source: Int J Mol Sci. 2026 Oct 18;27(20):9243. doi: 10.3390/ijms27209243 (PMC13705117; doi:10.3390/ijms27209243)
Supplement: Supplementary file 1 [file ijms-27-09243-s001.zip › Table SM.pdf]

Table S1: qPCR primers used for gene expression analysis

Genes

Sequence 5'-3'

β-actin – F GCAGAAGGAGATCACATCCCTGGC

β-actin – R CATTGCCGTCACCTTCACCGTTC

il-1β – f GGCAACTGTTCCTGAACTCAACTG

il-1β – r CCATTGAGGTGGAGAGCTTTCAGC

il-6 – f CCACTTCACAAGTCGGAGGCTT

il-6 – r CCAGCTTATCTGTTAGGAGA

il-4 – f TCTCTGCCAAGCAGGAATG

il-4 – r CAGTTTCCAGTCCCGGTATATG

nrxn-1 – f GAGCAGTAGCGATGAGATTAC

nrxn-1 – r ACTACCGCCGACATAGAA

ngln-3 – f CTGCTGACTCTTTCCCATTAT

ngln-3 – r CCTGCTCCACTAGTTCTTTG

il-10 – f CACAACCCCAATCGACTCCA

il-10 – r GGCAAGAAAAGTACCTCTTGCAT
